# Corneal Endothelial Morphology in Children with Type 1 Diabetes

**DOI:** 10.1155/2016/7319047

**Published:** 2016-06-26

**Authors:** Mohamed Anbar, Hatem Ammar, Ramadan A. Mahmoud

**Affiliations:** ^1^Department of Ophthalmology, Faculty of Medicine, Sohag University, Sohag 82524, Egypt; ^2^Department of Pediatrics, Faculty of Medicine, Sohag University, Sohag 82524, Egypt

## Abstract

*Aim*. To investigate corneal endothelial cell morphological in children with type 1 diabetes and to determine the systemic and local factors that contribute to these changes.* Methods*. One hundred sixty eyes of 80 children with type 1 diabetes and 80 eyes of 40 normal children as a control during the period from July 2015 to February 2016 underwent full clinical and ophthalmologic examination. We measured the central corneal thickness (CCT), endothelial cell density (ECD), ploymegathism, and pleomorphism using a noncontact specular microscope.* Results*. The mean age of the diabetic children was 8.22 ± 3.11 years. The mean duration of type 1 diabetes was 3.51 ± 2.23 years. The mean CCT was significantly higher: 537 ± 33.41 microns (right eye), in the diabetic group compared to the control group. The mean ECD in patients with type 1 diabetes was 3149.84 ± 343.75 cells/mm^2^ (right eye), and it was significantly lower than in the control group. Furthermore, pleomorphism was significantly lower 48.73 ± 5.43% (right eye), in the diabetic group compared to the control group. The mean polymegathism was significantly higher 37.96 ± 5.61% (right eye), in the diabetic group compared to the control group. All of these changes are significantly correlated only with the duration of diabetes.* Conclusions*. Diabetic children have thicker corneas, lower ECD, an increased polymegathism, and a decreased pleomorphism. The duration of diabetes is the factor that affects all of these changes. To what extent these changes affect visional function on long term needs to be investigated in further studies.

## 1. Introduction

Type 1 diabetes is a chronic illness characterized by the body's inability to produce insulin due to the autoimmune destruction of the beta cells in the pancreas. Onset most often occurs in childhood. Type 1 diabetes is the most common metabolic disease of childhood. About one in every 400–600 children and adolescents has type 1 diabetes. Its prevalence has increased over recent years. Diabetes mellitus is a systemic disease that alters the major metabolic pathway in the human body, affecting all organ systems [1–3].

Eye complications occur with long standing diabetes. Diabetic retinopathy is the most commonly investigated ocular complication [4]. However, morphologic and functional changes in the cornea have been studied less frequently in diabetic eyes. The corneal endothelium is a single layer of uniformly sized cells with a hexagonal shape. Their amount decreases by approximately 0.5%–0.6% (100–200 cells) per year [5]. The endothelial cell dysfunction is observed in myopia and in contact lens wearers [6, 7].

When corneal endothelial function decreases, corneal hydration and, consequently, the central corneal thickness (CCT) increase [8]. Many studies have shown that even minor changes in the morphology of the endothelial cells may manifest in the disturbances in the tightness of the endothelial barrier [5, 8, 9].

There are many reports concerning an analysis of the corneal endothelium in adults with type 1 and type 2 diabetes [10–13]. However, there are only some publications concerning an analysis of the cornea in type 1 diabetes in children [14, 15]. Prevention of the corneal endothelium dysfunction, its early detection, and immediate treatment are thus crucial, particularly if the problem concerns young patients. Therefore, the aim of this prospective cross-sectional study was to measure the central corneal thickness (CCT), endothelial cell density (ECD), coefficient of variation of cell size (polymegathism), and corneal cell variation in shape as a percentage of hexagonal cells (pleomorphism) using a noncontact specular microscope and to evaluate the local and systemic factors that may affect the corneal endothelium in this group of patients.

## 2. Materials and Methods

The current study was performed at the Pediatrics Diabetic outpatients clinic in the Pediatrics Department in cooperation with the Department of Ophthalmology, Faculty of Medicine, Sohag University, during the period from July 2015 to February 2016. Ethical approval for the study and the investigation was obtained from the Research Committee of Medical Faculty at Sohag University, and written informed consent was obtained from all parents of the children.

For the purpose of this study we examined corneal endothelial morphology in 160 eyes of 80 children with type 1 diabetes. We also examined 80 eyes of 40 normal children as a control. Children who used contact lenses or had previous ocular trauma, prior ocular surgery, intraocular inflammation, or refractive errors or who were taking topical medication were excluded from the study.

A complete history was taken for all diabetic patients including age, gender, and diabetes duration; the general examination included body mass index (BMI) and other systemic examinations; laboratory data collection included hemoglobin level and last glycosylated hemoglobin (HbA1C) level. Data from both eyes of every diabetic patient and the control children were collected in this study—a complete ophthalmological examination including visual acuity, slit lamp examination, intraocular pressure measurements, and fundus examination with a binocular indirect ophthalmoscopy. The corneal endothelium morphology, including central corneal thickness (CCT), endothelial cell density (ECD), corneal cell variation in size as percentage of abnormal size (corneal polymegathism), and corneal cell variation in shape as a percentage of hexagonal cells (corneal pleomorphism), was analyzed using a noncontact specular microscope (Topcon SP-1P, Tokyo, Japan). Endothelial cell density (ECD) was performed by asking the patients to look to the fixation target, when the pupil is displayed; operator taped the area around the pupil; the photographing head moves to display the pupil image and the alignment dot on the center of the screen. Alignment starts automatically and photographing is performed. Multiple images were taken with panorama view and the best quality image was analyzed. The best image quality was obtained when all cell borders, boundaries, and centers across a single image of the endothelium are distinct excluding the peripheral edges of the image and had a sufficient number of cells to count at least 50 and as many as 150 cells contiguous to each other. In this study the centers of 100 adjacent cells were marked and analyzed by built-in image analysis software.

Therefore, the aim of this study was to compare the corneal endothelial cell morphology and the central corneal thickness in diabetic and normal children and to correlate the abnormalities—if found—to risk factors such as age, sex, BMI, hemoglobin level, duration of diabetes, or metabolic control.

### 2.1. Statistical Analysis

Data was analyzed using STATA intercooled version 12.1. Quantitative data was represented as mean and standard deviation. Data was analyzed using Student's *t*-test to compare the means of two groups. Qualitative data was presented as a number and percentage and compared using chi-square test. Multivariate regression analyses were done to determine different eye parameters. *P* value was considered significant if it was less than 0.05.

## 3. Results

In this study, there were no abnormalities found in ophthalmological examination including visual acuity, slit lamp examination, intraocular pressure measurements, and fundus examination in type 1 diabetes patients or control group. Furthermore, as shown in Table 1, the corneal endothelial morphology in 160 eyes of 80 children with type 1 diabetes (32 (40%) boys and 48 (60%) girls) was examined. The age of the diabetic children ranged from 2 to 14 years (mean: 8.22 ± 3.11). The duration of type 1 diabetes ranged from 0.5 to 8 years (mean: 3.51 ± 2.23), which was subdivided into 30 (37.50%) children with a duration less than 2.5 years, 33 (41.25%) children with a duration of 2.5 to 5 years, and 17 (21.25%) children with a duration of more than 5 years. We also examined 80 eyes of 40 normal children as a control (14 (35%) boys and 26 girls (65%)); the age of the control group ranged from 3 to 13 years (mean: 7.83 ± 2.48).

As shown in Table 2, the range of HbA1C was 6.5% to 14.2% with a mean value of 8.33 ± 2.3%. There were 31 (38.75%) children with poor metabolic control (HbA1C, >8%), 27 (33.75%) children with moderate metabolic control (HbA1C, 7-8%), and 22 (27.50%) children with good metabolic control (HbA1C < 7%).

As shown in Table 3, a group of 80 children with type 1 diabetes was examined. The mean endothelial cell density (ECD) of the right eye in patients with diabetes was 3149.84 ± 343.75 cells/mm^2^, and it was significantly lower than that in the control group (3308.78 ± 99.33 cells/mm^2^) (*P* = 0.005). The mean endothelial cell density (ECD) of the left eye in patients with diabetes was 3142.13 ± 416.74 cells/mm^2^ and it was significantly lower than that in the control group (3315.25 ± 100.16 cells/mm^2^) (*P* = 0.01). The mean CCT of the right eye was 537 ± 33.41 microns in the diabetic group versus 504.7 ± 23.99 microns in the control group (*P* < 0.0001), while the mean CCT of the left eye was 539.91 ± 30.49 microns in the diabetic group versus 501.63 ± 15.77 microns in the control group (*P* < 0.0001).

As shown also in Table 3, the mean pleomorphism of the right eye was 48.73 ± 5.43% in the diabetic group versus 56.46 ± 9.64% in the control group (*P* < 0.0001), while the mean pleomorphism of the left eye was 49.67 ± 6.87% in the diabetic group versus 55.14 ± 10.27% in the control group (*P* < 0.003). Furthermore, the mean polymegathism of the right eye was 37.96 ± 5.61% in the diabetic group versus 35.55 ± 5.16% in the control group (*P* < 0.02). The mean polymegathism of the left eye was 36.45 ± 5.47% in the diabetic group versus 34.45 ± 3.03% in control group (*P* < 0.03).

As shown in Tables 4 and 5, the only factor affecting corneal endothelium morphology in children with type 1 diabetes was the duration of diabetes. We determined the odds ratio (95% confidence intervals) and did not find any correlation between ECD, CCT, pleomorphism, polymegathism, and the following variables: the age of the patients, gender, HbA1C level, BMI, hemoglobin level, and presence of diabetic retinopathy.

**Figure 1:**
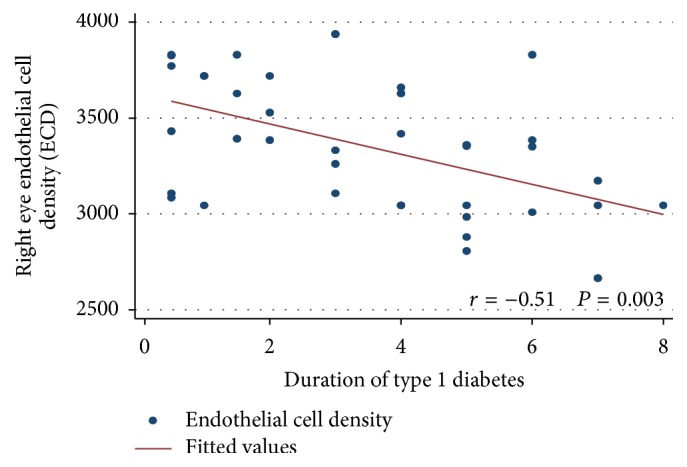
Correlation between the right endothelial cell density (ECD) and duration of type 1 diabetes in years.

**Figure 2:**
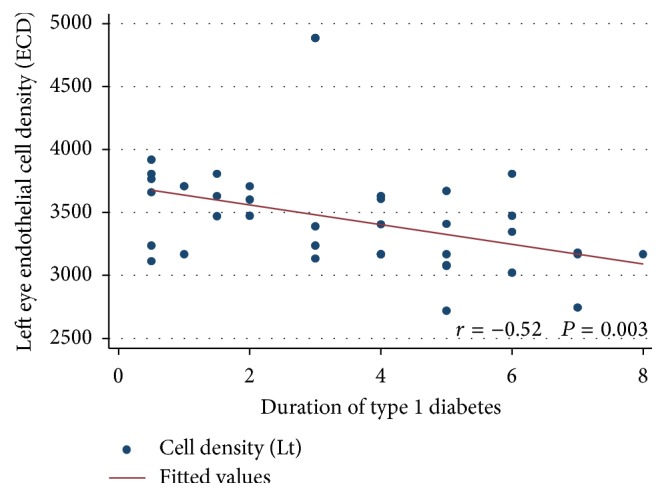
Correlation between the left endothelial cell density (ECD) and duration of type 1 diabetes in years.

As shown in Table 4, there was a positive correlation between right CCT, left CCT, and duration of diabetes in years (odds ratio (95% confidence intervals) = 6.53 (2.19 : 10.88) and 5.22 (1.20 : 9.25), *P* value = 0.004 and 0.01, resp.). Moreover, as shown in Figures 1 and 2, there was a negative correlation between the mean right ECD, left ECD, and the duration of diabetes in years (*r* = −0.51, *P* = 0.003, and *r* = −0.52, *P* = 0.003, resp.).

Furthermore, as shown in Table 5, there was a negative correlation between right corneal pleomorphism, left corneal pleomorphism, and the duration of diabetes in years (odds ratio (95% confidence intervals) = −1.11 (−2.32 : 0.19) and −0.66 (−1.8 : −0.41), *P* value = 0.04 and 0.02, resp.). Moreover, there was a positive correlation between the right corneal polymegathism, left corneal polymegathism, and the duration of diabetes in years (odds ratio (95% confidence intervals) = 0.61 (0.23 : 1.81) and 5.22 (1.20 : 9.25), *P* value = 0.04 and 0.01, resp.).

## 4. Discussion

With the progress of the techniques and instrumentation used in the evaluation of the corneal endothelial cells from the contact to noncontact specular microscopy, we were encouraged to evaluate the corneal endothelial cell morphology in children with certain eye and systemic diseases compared to normal children. Of course, diabetes mellitus is considered one of the most systemic diseases affecting the eye in general, and, in particular, we opened a window in this study to see the effect of type 1 diabetes on the corneal endothelial cell count and morphology in children. We found that the central cornea of type 1 diabetes is generally thicker than that of normal persons and had lower corneal endothelial cell density, lower hexagonality, and higher CV of cell size compared to nondiabetic children.

Several studies have been done to evaluate the corneal endothelial cells in diabetic adults [16–20]. Most of these studies agreed that diabetic corneas tend to be thicker and had more polymegathism and less pleomorphism, and they correlated these changes to the duration of the diabetes and/or to the status of the metabolic control. However, there have been very limited studies concerning the changes that occur in corneal endothelium of diabetic children, and the cause for the scant studies is not clear but may be explained by the fact that the noncontact specular microscope used in these measurements has only recently become available.

In our study we evaluated the changes that may occur in the corneal endothelium of diabetic children and compared these changes with the endothelium of nondiabetic children. We tried to find the risk factors that may contribute to these changes. For this reason, we evaluated four parameters of the corneal endothelium including the CCT, ECD, pleomorphism, and polymegathism. We have established that the mean CCT is higher in children with diabetes than in normal children and mean ECD is lower.

Similar results were obtained by Urban et al. [14] who examined 123 eyes of type 1 diabetic children and 124 eyes of nondiabetic children. The mean age of diabetic children was 15.34 ± 3.06 years versus 14.58 ± 2.01 years in the control group. The mean duration of diabetes was 8.02 ± 3.9 years. They found a reduction of the ECD by 18% compared to the control group. They also found that the mean CCT was 550 ± 30 microns in the diabetic group versus 530 ± 33 microns in the control group.

In a study done by Tiutiuca [15] in which the CCT was measured for 100 children with type 1 diabetes (study group) and 100 healthy children (control group), the average CCT in diabetic children was 541 ± 30 microns for the right eye and 538 ± 32 microns for the left eye; in the control group it was 528 ± 33 microns for the right eye and 526 ± 30 microns for the left eye. He concluded that diabetic children have a significantly increased CCT when compared with nondiabetic children. The same results were obtained in another study by Urban et al. [21] who also evaluated corneal thickness in diabetic and nondiabetic children. They found a significant increase in the CCT in diabetic children when compared to nondiabetic controls.

Furthermore, a study was done by Módis Jr. et al. [22] who compared the effect of type 1 and type 2 diabetes on the corneal endothelial cell morphology. There was a statistically significant decreased ECD in type 1 diabetes in comparison with healthy subjects, but in type 2 diabetes no significant difference was found in the evaluated values. The mean age of type 1 diabetes in Módis Jr. et al.'s study was 40.97 ± 15.46 compared to 8.22 ± 3.11 in our study. Also, Roszkowska et al. [8], after examining 75 adults with type 1 and type 2 diabetes, noted that the ECD decreased by 5% in type 2 diabetes and by 11% in type 1 diabetes when compared with healthy persons.

In a relatively old study done by Schultz et al. [23], a significantly higher rate of cell loss in type 1 diabetes was detected, resulting in a significant decrease in ECD in the fourth and fifth decades. On the other hand, several studies have evaluated the effect of type 2 diabetes on corneal endothelium [18, 20, 24–26]. Most of these studies agreed that the ECD is reduced and the cornea is thicker in type 2 diabetes.

Different results were obtained by Larsson et al. [13] who examined 49 patients with type 1 diabetes and 60 patients with type 2 diabetes. They concluded that neither type 1 diabetes patients nor type 2 diabetes patients differed from their controls in ECD but type 1 diabetes patients had thicker corneas. Furthermore, Furuse et al. [27] did not demonstrate significant changes in mean ECD in diabetic subjects, but they only examined patients with type 2 diabetes.

In order to determine the systemic and local risk factors affecting the corneal endothelial morphology in children with type 1 diabetes, we determined the odds ratio (95% confidence intervals) and we did not find any correlation between ECD and CCT with the following variables: the age of the patients, gender, HbA1C level, BMI, and hemoglobin level. In this study the only factor affecting corneal endothelium morphology in type 1 diabetes patients was the duration of diabetes. The same results were obtained by Urban et al. [14] who demonstrated a significant correlation between ECD and the duration of diabetes in children. Also, Busted et al. [28] found a correlation between the lower ECD and the higher CCT in type 1 diabetes patients with the duration of the disease, but the decrease per year was of the same magnitude as the normal age dependence.

Furthermore, Calvo-Maroto et al. [18] found in a study on adult patients with type 2 diabetes significant alteration in corneal structure and function in the long term diabetes of more than 10 years with poor glycemic control. Moreover, Lee et al. [29] found a correlation between the diabetes duration with only the CCT but not with the ECD. There were different studies that did not find any correlated risk factors with the endothelial cell changes in diabetic patients, but these studies were conducted in type 2 diabetes [24–26, 30, 31].

In this study, there were no cases with diabetic retinopathy. However, Siribunkum et al. [32] in adult diabetic patients found a correlation between the severity of diabetic retinopathy and the reduction in ECD, while the CCT was correlated only with the duration of the disease. They also found that the corneal changes were not correlated with glycemic control. Moreover, Módis Jr. et al. [22] found that HbA1C is inversely correlated with ECD in type 1 diabetes, while in type 2 diabetes no changes occurred. Ziadi et al. [33] did not detect any relation between the level of HbA1C and the condition of the corneal endothelium.

Furthermore, we have established a significant increase in polymegathism and decrease in pleomorphism in children with type 1 diabetes. Also, we found that the only risk correlated with the changes in the polymegathism and pleomorphism is the duration of the disease. The same results were obtained by Módis Jr. et al. [22]. Moreover, Lee et al. [29] found that diabetic subjects had less hexagonality and a more irregular cell size of the corneal endothelium than did the controls. Like our study, they correlated the corneal morphological abnormalities, especially the coefficient of variation in cell size, to the duration of diabetes.

Inoue et al. [30] investigated the corneal endothelial structure in 99 patients with type 2 diabetes and in 97 nondiabetic patients. They found an increase in the polymegathism in diabetic patients. However, the pleomorphism in diabetic patients was not significantly different from that in nondiabetic patients. Siribunkum et al. [32] concluded in their study that the diabetic corneas had more polymegathism and less pleomorphism, though this was not statistically significant. The duration of diabetes was correlated significantly with these corneal changes.

Also Larsson et al. [13] noticed significant variability in endothelial cell size and a significant decrease in endothelial cell hexagonality in type 1 diabetes patients when compared to their controls. They concluded that the corneas of patients with type 1 diabetes exhibit abnormalities in endothelial cell morphologic characteristics. The changes resemble those that occur with aging in normal subjects.

Matsuda et al. [31] found that, in type 2 diabetes, when compared to age-matched nondiabetic controls, all diabetic groups demonstrated significant increases in cell size and shape variability. Unlike our study, none of the endothelial morphologic parameters were found to correlate with the duration of diabetes or glycemic control. On the other hand, their study was conducted on patients with type 2 diabetes.

## 5. Conclusion

The conclusion of this study is that diabetic children have thicker corneas, lower ECD, an increase in cell size variability (CV), and a decrease in the hexagonal cells. The duration of diabetes is the factor that affects all of these changes. To what extent these changes affect visional function on long term needs to be investigated in further studies.

## Figures and Tables

**Table 1:** Criteria of studied populations.

Patient characteristics	Type 1 diabetes	Healthy controls	*P* value
Age (years)			
Mean (SD)	8.22 ± 3.11	7.83 ± 2.48	0.48
Median (range)	9 (2–14)	8 (3–13)	
Gender			
Females (median and range)	48 (60.00%)	26 (65.00%)	0.60
Males (median and range)	32 (40.00%)	14 (35.00%)	
Weight (kg)			
Mean (SD)	28.34 ± 9.73	26.05 ± 6.36	0.18
BMI			
Mean (SD)	17.18 ± 2.81	17.61 ± 1.57	0.38
Hemoglobin (g/dL)			
Mean (SD)	12.37 ± 1.04	12.54 ± 0.51	0.35
HbA1C in percentage			
Mean (SD)	8.33% ± 2.31	4.33% ± 0.83	<0.0001

BMI: body mass index; HbA1C: glycosylated hemoglobin.

**Table 2:** The duration of type 1 DM in years and the degree of metabolic control represented by HbA1C percentage.

Patient characteristics	Summary statistics
Duration of type 1 DM/years	
<2.5 years	30 (37.50%)
2.5–5.0 years	33 (41.25%)
>5 years	17 (21.25%)

HbA1C	
<7%	22 (27.50%)
7-8%	27 (33.75%)
>8%	31 (38.75%)

HbA1C: glycosylated hemoglobin.

**Table 3:** Comparison between type 1 diabetes patients and healthy controls as regards corneal endothelial morphology.

Patient characteristics	Type 1 diabetes	Healthy controls	*P* value
Right central corneal thickness (microns)			
Mean (SD)	537 ± 33.41	504.7 ± 23.99	<0.0001
Right endothelial cell density (cells/mm^2^)			
Mean (SD)	3149.84 ± 343.75	3308.78 ± 99.33	0.005
Right pleomorphism %			
Mean (SD)	48.73 ± 5.43	56.46 ± 9.64	<0.0001
Right polymegathism %			
Mean (SD)	37.96 ± 5.61	35.55 ± 5.16	0.02
Left central corneal thickness (microns)			
Mean (SD)	539.91 ± 30.49	501.63 ± 15.77	<0.0001
Left endothelial cell density (cells/mm^2^)			
Mean (SD)	3142.13 ± 416.74	3315.25 ± 100.16	0.01
Left pleomorphism %			
Mean (SD)	49.67 ± 6.87	55.14 ± 10.27	0.003
Left polymegathism			
Mean (SD)	36.45 ± 5.47	34.45 ± 3.03	0.03

Pleomorphism: percentage of normal hexagonal cells; polymegathism: percentage of abnormal corneal cell size.

**Table 4:** Factors affecting corneal endothelial morphology of type 1 diabetes as regards central corneal thickness and endothelial cell density.

	Odds ratio (95% confidence intervals)	*P* value
Factors affecting right central corneal thickness		
Age	−3.68 (−7.84 : 0.47)	0.08
Male versus female	5.51 (−9.90 : 20.93)	0.47
Duration of type 1 diabetes/years	6.53 (2.19 : 10.88)	0.004
BMI	−0.52 (−3.66 : 2.63)	0.74
Hemoglobin	6.30 (−5.14 : 17.75)	0.28
HbA1C	2.13 (−2.09 : 6.35)	0.32
Factors affecting left central corneal thickness		
Age	−3.15 (−6.99 : 0.69)	0.10
Male versus female	3.86 (−10.41 : 18.13)	0.59
Duration of type 1 diabetes/years	5.22 (1.20 : 9.25)	0.01
BMI	−0.63 (−3.54 : 2.28)	0.67
Hemoglobin	2.96 (−7.64 : 13.56)	0.58
HbA1C	0.03 (−3.87 : 3.94)	0.98
Factors affecting right endothelial cell density		
Age	0.18 (−0.49 : 0.79)	0.61
Male versus female	−4.79 (−7.28 : 2.31)	0.35
Duration of type 1 diabetes/years	−35.93 (−67.38 : −4.48)	0.03
BMI	−6.69 (−29.41 : 16.02)	0.56
Hemoglobin	54.34 (−28.46 : 137.13)	0.20
HbA1C	4.11 (−6.60 : 7.62)	0.31
Factors affecting left endothelial cell density		
Age	0.16 (−0.47 : 0.80)	0.62
Male versus female	−4.35 (−6.70 : 2.00)	0.34
Duration of type 1 diabetes/years	−32.93 (−65.38 : −6.48)	0.03
BMI	0.03 (−0.45 : 0.51)	0.90
Hemoglobin	−1.21 (−2.96 : 0.53)	0.17
HbA1C	−0.04 (−0.68 : 0.61)	0.91

BMI: body mass index; HbA1C: glycosylated hemoglobin.

**Table 5:** Factors affecting corneal endothelial morphology of type 1 diabetes as regards corneal pleomorphism and polymegathism.

	Odds ratio (95% confidence intervals)	*P* value
Factor affecting right corneal pleomorphism		
Age	0.59 (−0.57 : 1.74)	0.31
Male versus female	5.33 (−1.06 : 9.61)	0.15
Duration of type 1 diabetes/years	−1.11 (−2.32 : 0.19)	0.04
BMI	−0.64 (−0.151 : 0.23)	0.15
Hemoglobin	−0.11 (−3.29 : 3.06)	0.94
HbA1C	0.26 (−0.91 : 1.43)	0.66
Factor affecting left corneal pleomorphism		
Age	−1.24 (−2.36 : 0.12)	0.30
Male versus female	7.40 (−3.22 : 11.56)	0.18
Duration of type 1 diabetes/years	−0.66 (−1.8 : −0.41)	0.02
BMI	−0.22 (−0.62 : 1.07)	0.60
Hemoglobin	2.45 (−0.64 : 5.54)	0.12
HbA1C	−0.27 (−1.41 : 0.87)	0.65
Factor affecting right corneal polymegathism		
Age	−0.28 (−0.91 : 0.35)	0.38
Male versus female	−4.02 (−6.36 : 1.68)	0.14
Duration of type 1 diabetes/years	0.61 (0.23 : 1.81)	0.04
BMI	0.43 (−0.40 : 0.91)	0.11
Hemoglobin	0.51 (−1.23 : 2.24)	0.56
HbA1C	−0.36 (−0.99 : 0.28)	0.27
Factor affecting left corneal polymegathism		
Age	0.16 (−0.47 : 0.79)	0.62
Male versus female	−4.35 (−6.70 : 2.01)	0.45
Duration of type 1 diabetes/years	5.22 (1.20 : 9.25)	0.01
BMI	0.03 (0.45 : 0.51)	0.90
Hemoglobin	−1.21 (−2.96 : 0.53)	0.17
HbA1C	−0.04 (−0.68 : 0.61)	0.91

BMI: body mass index; HbA1C: glycosylated hemoglobin.
